# A Cell System for Phenotypic Screening of Modifiers of SMN2 Gene Expression and Function

**DOI:** 10.1371/journal.pone.0071965

**Published:** 2013-08-15

**Authors:** Darrick K. Li, Sarah Tisdale, Jorge Espinoza-Derout, Luciano Saieva, Francesco Lotti, Livio Pellizzoni

**Affiliations:** 1 Center for Motor Neuron Biology and Disease, Columbia University, New York, New York, United States of America; 2 Department of Pathology and Cell Biology, Columbia University, New York, New York, United States of America; International Centre for Genetic Engineering and Biotechnology, Italy

## Abstract

Spinal muscular atrophy (SMA) is an inherited neurodegenerative disease caused by homozygous inactivation of the *SMN1* gene and reduced levels of the survival motor neuron (SMN) protein. Since higher copy numbers of the nearly identical *SMN2* gene reduce disease severity, to date most efforts to develop a therapy for SMA have focused on enhancing SMN expression. Identification of alternative therapeutic approaches has partly been hindered by limited knowledge of potential targets and the lack of cell-based screening assays that serve as readouts of SMN function. Here, we established a cell system in which proliferation of cultured mouse fibroblasts is dependent on functional SMN produced from the *SMN2* gene. To do so, we introduced the entire human *SMN2* gene into NIH3T3 cell lines in which regulated knockdown of endogenous mouse Smn severely decreases cell proliferation. We found that low *SMN2* copy number has modest effects on the cell proliferation phenotype induced by Smn depletion, while high *SMN2* copy number is strongly protective. Additionally, cell proliferation correlates with the level of SMN activity in small nuclear ribonucleoprotein assembly. Following miniaturization into a high-throughput format, our cell-based phenotypic assay accurately measures the beneficial effects of both pharmacological and genetic treatments leading to SMN upregulation. This cell model provides a novel platform for phenotypic screening of modifiers of SMN2 gene expression and function that act through multiple mechanisms, and a powerful new tool for studies of SMN biology and SMA therapeutic development.

## Introduction

Spinal muscular atrophy (SMA) is an autosomal recessive neurodegenerative disease characterized by loss of motor neurons in the anterior horn of the spinal cord and skeletal muscle atrophy [1]. SMA is caused by reduced levels of the survival motor neuron (SMN) protein, an evolutionarily conserved and ubiquitously expressed protein essential for viability [2], [3]. SMN exists in a macromolecular complex with functions in the assembly of the small nuclear ribonucleoproteins (snRNPs) of the RNA splicing machinery and possibly other RNA-protein complexes [4]–[6]. In animal models of SMA, the disruption of snRNP biogenesis induced by SMN deficiency decreases snRNP levels [7]–[9] and causes splicing defects in genes that contribute to motor system dysfunction [10]–[12].

The human genome contains two genes that code for the SMN protein, *SMN1* and *SMN2*, with multiple copies of *SMN2* present [13]. SMA patients have homozygous loss or mutations of the *SMN1* gene and retention of at least one copy of *SMN2*. Although the two *SMN* genes are nearly identical, a C to T transition in exon 7 of *SMN2* disrupts splicing regulatory elements resulting mainly in the production of transcripts lacking exon 7 (SMNΔ7) with only a small proportion encoding full-length SMN [14]–[17]. SMN2 exon 7 skipping creates a destabilizing element responsible for the rapid degradation of the SMNΔ7 protein [18]–[20]. As a consequence, reduced levels of full-length SMN protein produced from the *SMN2* gene, while sufficient to prevent embryonic lethality, are not able to fully compensate for the loss of *SMN1* resulting in motor neuron disease.

There is a direct connection between SMN protein levels and SMA severity as higher *SMN2* gene copy number correlates with milder forms of the disease in patients [21]–[23]. Thus, most efforts in developing SMA therapeutics have focused on methods to increase SMN protein levels. These include activation of the *SMN2* promoter, enhancing inclusion of exon 7 in *SMN2*-derived transcripts, increasing the stability of SMN mRNA and protein, or restoring SMN expression through gene therapy [24]–[27]. In agreement with the viability of these approaches, injection of AAV vectors encoding full-length SMN in mouse models of SMA resulted in remarkable correction of lifespan and motor function [28]–[31]. Similarly, robust phenotypic benefit in SMA mice was accomplished by promoting *SMN2* exon 7 inclusion using antisense oligonucleotides targeting intronic splicing silencers [32], [33]. Small chemical compounds that increase SMN expression are also being investigated for SMA therapy. The use of histone deacetylase (HDAC) inhibitors has been shown to result in phenotypic improvement in SMA mice through *SMN2* transcriptional upregulation [34], [35]. Additional inducers of SMN expression have been identified in high-throughput chemical screens [36]–[40]. C5-substituted quinazolines [37], the most clinically advanced therapeutic candidates emerged from these screens, potently inhibit the activity of the scavenger decapping enzyme DcpS [41] and improve survival and motor phenotype in SMA mice [42]–[44]. Although these candidate therapeutics are now entering clinical trials, SMA remains the most frequent genetic cause of infant mortality worldwide for which no effective treatment is currently available.

Ongoing efforts to address the high unmet clinical need in SMA would benefit from increased knowledge of basic SMN biology and disease mechanisms as well as identification of alternative therapeutic approaches. In addition to upregulation of SMN expression, strategies aiming to enhance SMN function or to correct downstream effects of SMN deficiency might provide new avenues for SMA therapy. Accordingly, there is evidence that SMN function is regulated both *in vivo* and *in vitro* [45], [46] and that targeting downstream defects induced by SMN deficiency without increasing SMN levels can be beneficial in animal models of SMA [10], [11], [47]–[50]. However, progress towards the development of alternative therapeutic strategies for SMA has generally been hindered by lack of suitable targets as well as screening platforms that supply readouts of SMN function and downstream SMN-dependent events. In an effort to address this shortcoming, we developed a novel cell-based system for phenotypic screening of chemical or genetic modifiers that may increase SMN expression and function through multiple mechanisms of action. To do so, we have taken advantage of a previously characterized mouse NIH3T3 cell line in which regulated knockdown of endogenous mouse Smn triggers a severe cell proliferation defect [11], providing a direct phenotypic readout of SMN function. We introduced the human *SMN2* gene into this cell line, generating a system where cell proliferation is sensitive to changes in functional SMN levels produced from *SMN2*. We further miniaturized and validated a cell-based phenotypic assay in 96-well format amenable to high-throughput chemical and genetic screens. Our cell model system provides a novel tool for the discovery of cellular factors and genetic networks that control SMN biology in mammalian cells, which will not only increase our knowledge of the basic biology of SMN but also contribute to SMA therapeutic development.

## Results

### Development of Human SMN2-containing NIH3T3 Cell Lines with Regulated Knockdown of Endogenous Smn

**Figure 1:**
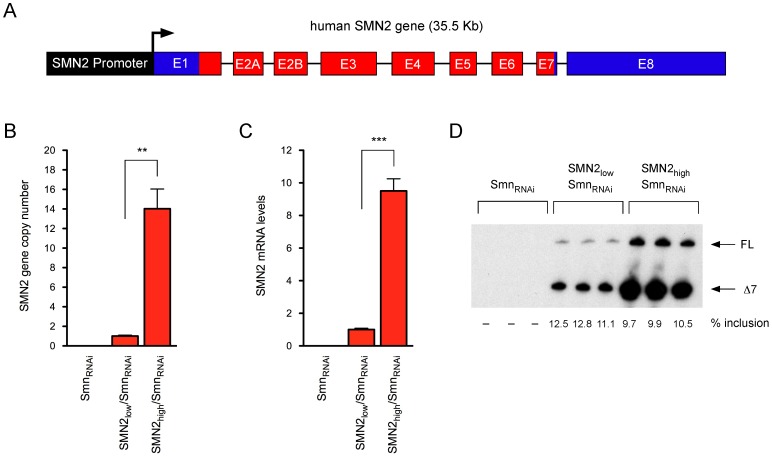
Development of *SMN2*-containing NIH3T3 cell lines with inducible knockdown of endogenous Smn. (A) Schematic representation of the exon-intron structure of the human *SMN2* gene used to establish NIH3T3-SMN2/Smn_RNAi_ cell lines (introns not drawn to scale). (B) Analysis of *SMN2* gene copy number in the indicated NIH3T3 cell lines by qPCR. Data were normalized to the *Gapdh* gene and expressed relative to the NIH3T3-SMN2_low_/Smn_RNAi_ cell line. Data are represented as mean and SEM (n = 3; ** = p<0.01; t-test). (C) Analysis of total SMN2 mRNA levels in the indicated NIH3T3 cell lines by RT-qPCR. Data were normalized to Gapdh mRNA and expressed relative to the NIH3T3-SMN2_low_/Smn_RNAi_ cell line. Data are represented as mean and SEM (n = 3; *** = p<0.001, t-test). (D) Analysis of SMN2 exon 7 splicing in the indicated NIH3T3 cell lines by radioactive RT-PCR. The levels of exon 7 inclusion are shown at the bottom.

We recently established and characterized an NIH3T3-Smn_RNAi_ cell line with drug-inducible, RNAi-mediated knockdown of endogenous mouse Smn [11], [12]. In these cells, addition of doxycycline causes Smn depletion and subsequent, severe cell proliferation defects that can be corrected by transgenic expression of RNAi-resistant human SMN [11]. We sought to modify this model system so that cell proliferation would be dependent on SMN levels produced by the human *SMN2* gene. To do so, the 35.5 kb BamHI fragment corresponding to the genomic region encompassing the *SMN2* gene (Figure 1A), previously used to generate SMA mice [51], was cloned into a cosmid vector containing a neomycin selection cassette under the control of the SV40 promoter and the resulting construct was transfected into NIH3T3-Smn_RNAi_ cells. Several neomycin-resistant stable cell lines were isolated through antibiotic selection and cloning by limiting dilution in 96-well plates. Here, we describe the characterization of two representative NIH3T3-SMN2/Smn_RNAi_ cell lines with either low or high *SMN2* copy number. Genomic DNA from these NIH3T3 cell lines was isolated and the relative *SMN2* gene copy number was determined by quantitative PCR with human SMN-specific primers. NIH3T3-SMN2_high_/Smn_RNAi_ cells contained more than tenfold the number of *SMN2* copies present in NIH3T3-SMN2_low_/Smn_RNAi_ cells (Figure 1B). Consistent with this, RT-qPCR analysis with human-specific SMN primers showed that in the absence of doxycycline NIH3T3-SMN2_high_/Smn_RNAi_ cells expressed approximately ten times the amount of total SMN2 mRNA compared to NIH3T3-SMN2_low_/SMN_RNAi_ cells (Figure 1C). Importantly, radioactive RT-PCR analysis revealed the expected pattern of exon 7 splicing regulation of the *SMN2* gene in both cell lines (Figure 1D). Irrespective of the marked difference in the overall levels SMN2 expression, the majority of SMN2 transcripts lacked exon 7 (SMNΔ7) and only ∼10% were full-length SMN mRNA.

**Figure 2:**
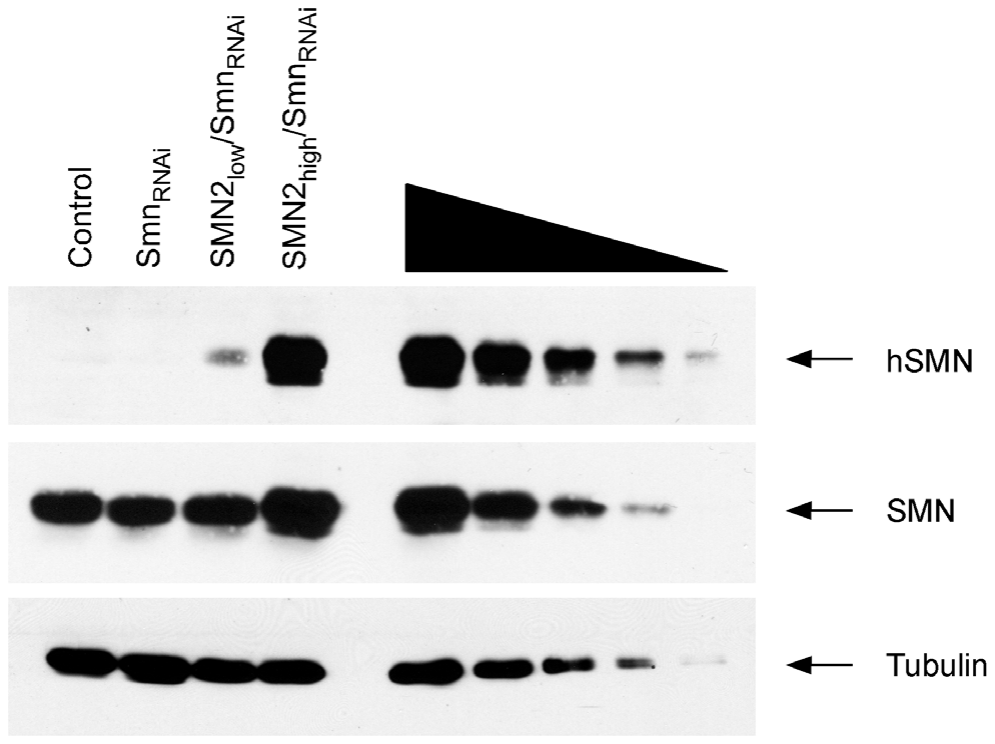
Analysis of human SMN protein levels in NIH3T3-SMN2/Smn_RNAi_ cell lines. Western blot of equal amounts of proteins from wild-type (Control), NIH3T3-Smn_RNAi_, NIH3T3-SMN2_low_/Smn_RNAi_ and NIH3T3-SMN2_high_/Smn_RNAi_ cell lines with monoclonal antibodies against the indicated proteins. Monoclonal antibodies specific to human SMN (hSMN) or both mouse and human SMN (SMN) were used. A two-fold serial dilution of the extract from NIH3T3-SMN2_high_/Smn_RNAi_ cells is shown on the right.

**Figure 3:**
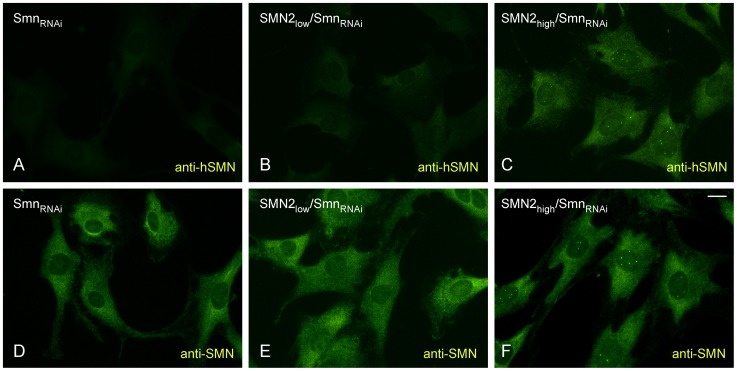
Subcellular localization of the human SMN protein in NIH3T3-SMN2/Smn_RNAi_ cell lines. Indirect immunofluorescence and confocal microscopy analysis of NIH3T3-Smn_RNAi_ (A and D), NIH3T3-SMN2_low_/Smn_RNAi_ (B and E) and NIH3T3-SMN2_high_/Smn_RNAi_ (C and F) cell lines using monoclonal antibodies specific to human SMN (hSMN, panels A–C) or both mouse and human SMN (SMN, panels D–F). Scale bar, 10 µm.

Next, we investigated the expression and subcellular localization of the human SMN protein in NIH3T3-SMN2/Smn_RNAi_ cells. In agreement with the *SMN2* gene copy number and mRNA expression levels, Western blot analysis with an antibody that specifically detects human SMN revealed that in the absence of doxycycline NIH3T3-SMN2_high_/Smn_RNAi_ cells express approximately tenfold more SMN than NIH3T3-SMN2_low_/Smn_RNAi_ cells (Figure 2). Human SMN protein was not detected in either wild-type or NIH3T3-Smn_RNAi_ cells that do not contain the *SMN2* gene, confirming the specificity of the antibodies. We then carried out immunofluorescence experiments with a human SMN-specific antibody (anti-hSMN) or an antibody that recognizes both mouse and human SMN proteins (anti-SMN) in NIH3T3 cell lines cultured without doxycycline. Analysis in NIH3T3-Smn_RNAi_ cells showed no staining with anti-hSMN but strong cytoplasmic and weak nuclear staining with anti-SMN (Figure 3A and 3D). As expected, staining with anti-hSMN was weak in NIH3T3-SMN2_low_/Smn_RNAi_ cells (Figure 3B) and strong in NIH3T3-SMN2_high_/Smn_RNAi_ cells (Figure 3C), and revealed a similar subcellular distribution to that of endogenous mouse Smn. Noticeably, SMN localization in nuclear foci known as Gems [52] while rare in wild-type NIH3T3 cells was frequently observed in NIH3T3-SMN2_high_/Smn_RNAi_ cells, possibly due to higher SMN levels.

**Figure 4:**
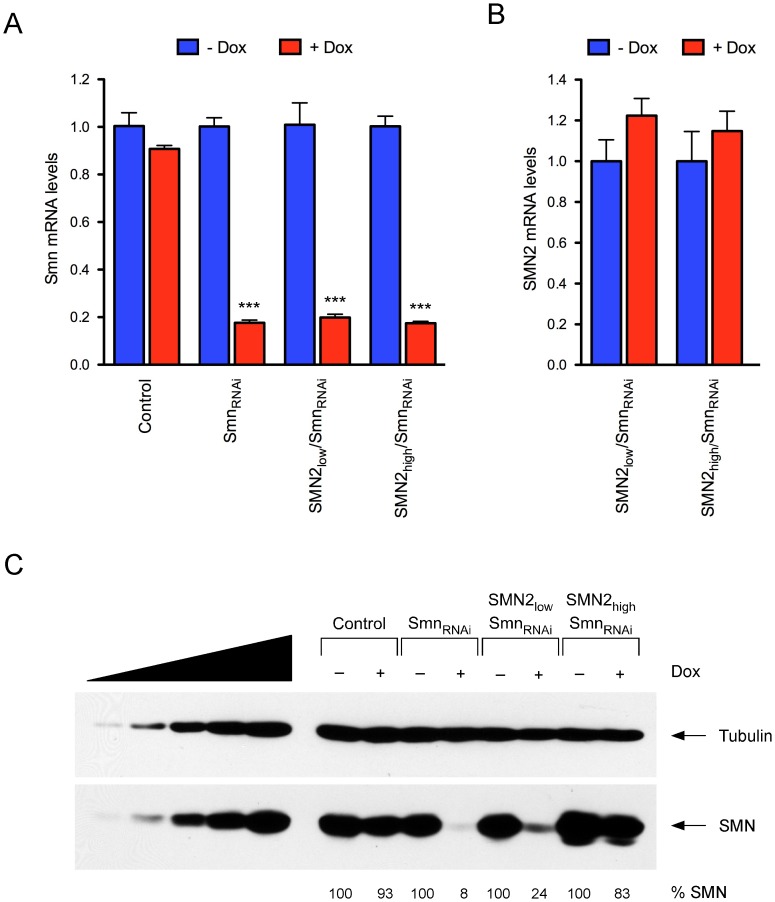
Inducible knockdown of endogenous mouse Smn in NIH3T3-SMN2/Smn_RNAi_ cell lines. (A) RT-qPCR analysis of mouse Smn mRNA levels in wild-type (Control), NIH3T3-Smn_RNAi_, NIH3T3-SMN2_low_/Smn_RNAi_ and NIH3T3-SMN2_high_/Smn_RNAi_ cell lines cultured with and without Dox for 7 days. Data in Dox-treated cells were normalized to those in untreated cells and represented as mean and SEM (n = 3; *** = p<0.001; t-test). (B) RT-qPCR analysis of human SMN2 mRNA levels in NIH3T3-SMN2_low_/Smn_RNAi_ and NIH3T3-SMN2_high_/Smn_RNAi_ cell lines cultured with and without Dox for 7 days. Data in Dox-treated cells were normalized to those in untreated cells and represented as mean and SEM. (C) Western blot analysis of equal amounts of proteins from wild-type (Control), NIH3T3-Smn_RNAi_, NIH3T3-SMN2_low_/Smn_RNAi_ and NIH3T3-SMN2_high_/Smn_RNAi_ cell lines cultured with and without Dox for 7 days using monoclonal antibodies against the indicated proteins. For each cell line, SMN levels in Dox-treated relative to untreated cells are shown at the bottom.

Lastly, we investigated whether the capacity for doxycycline-inducible, RNAi-mediated knockdown of endogenous Smn characteristic of parental NIH3T3-Smn_RNAi_ cells was retained in the newly established NIH3T3-SMN2/Smn_RNAi_ cell lines. We first analyzed the effects of doxycycline on the expression levels of mouse Smn mRNA by RT-qPCR. As expected [11], doxycycline-treated NIH3T3-Smn_RNAi_ cells had strongly decreased levels of Smn mRNA compared to untreated cells and doxycycline had no effects in wild-type NIH3T3 cells (Figure 4A). Doxycycline caused a reduction in Smn mRNA levels in both NIH3T3-SMN2_low_/Smn_RNAi_ and NIH3T3-SMN2_high_/Smn_RNAi_ cells similar to that in NIH3T3-Smn_RNAi_ cells, demonstrating that these cells preserved the ability for inducible knockdown of Smn mRNA. Consistent with the shRNA specific targeting of mouse Smn mRNA, human SMN2 mRNA levels were unaffected by RNAi induction with doxycycline in NIH3T3-SMN2/Smn_RNAi_ cells (Figure 4B). We then carried out Western Blot analysis to determine the levels of SMN protein expression following knockdown of endogenous Smn. In agreement with Smn mRNA levels and our previous studies [11], [12], doxycycline treatment of NIH3T3-Smn_RNAi_ cells for 7 days strongly reduced SMN protein levels to approximately 10% of normal, but had no effect in wild-type NIH3T3 cells (Figure 4C). Importantly, the amounts of SMN in doxycycline-treated NIH3T3-SMN2_low_/Smn_RNAi_ (∼20%) and NIH3T3-SMN2_high_/Smn_RNAi_ cells (∼80%) relative to untreated cells were consistent with the levels of human SMN mRNA expressed from the *SMN2* gene. Thus, we developed NIH3T3 cell lines with regulated knockdown of endogenous Smn that express either low or high levels of human SMN from the *SMN2* gene.

### Cell Proliferation Correlates with SMN2 Gene Expression and Function in Smn-deficient NIH3T3-SMN2/Smn_RNAi_ Cells

**Figure 5:**
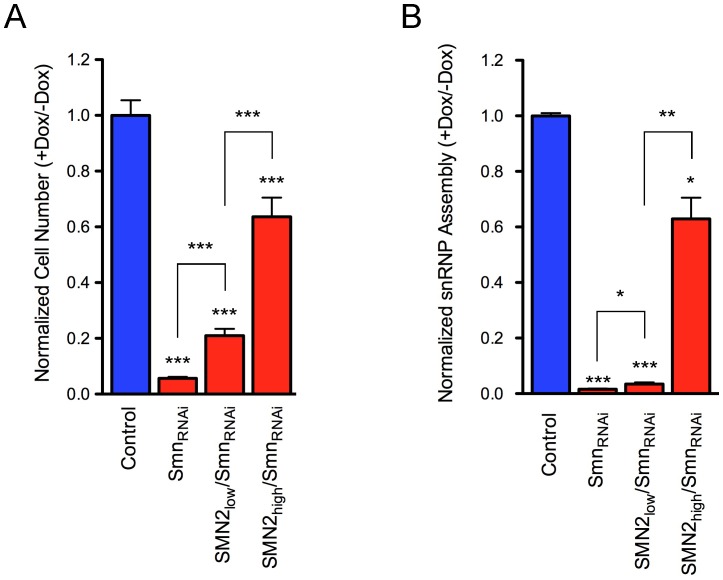
Effect of human SMN2 expression on proliferation and snRNP assembly in Smn-deficient NIH3T3 cell lines. (A) Analysis of cell proliferation in wild-type (Control), NIH3T3-Smn_RNAi_, NIH3T3-SMN2_low_/Smn_RNAi_ and NIH3T3-SMN2_high_/Smn_RNAi_ cell lines cultured with and without doxycycline for 7 days. For each cell line, the cell number ratio of Dox-treated cells versus untreated cells is expressed relative to that of wild-type cells, which is set to 1. Data are represented as mean and SEM (n≥3; *** = p<0.001; one-way ANOVA). (B) Analysis of U1 snRNP assembly in NIH3T3 cell lines. *In vitro* snRNP assembly experiments were carried out with radioactive U1 snRNA and extracts from wild-type NIH3T3 (Control), NIH3T3-Smn_RNAi_, NIH3T3-SMN2_low_/Smn_RNAi_ and SMN2_high_/Smn_RNAi_ cells cultured with and without doxycycline for 7 days. For each cell line, the amounts of immunoprecipitated U1 snRNA were quantified and the RNA ratio in Dox-treated cells versus untreated cells is expressed relative to that of wild-type cells, which is set to 1. Data are represented as mean and SEM (n≥3; * = p<0.05; ** = p<0.01; *** = p<0.001; one-way ANOVA).

SMN deficiency elicits a severe cell proliferation phenotype in NIH3T3 cells [11]. Smn-deficient NIH3T3-Smn_RNAi_ cells display decreased proliferation after 3 days of doxycycline treatment and become growth arrested at day 5, entering a quiescent proliferative state that could last for many days without significant cell death [11]. While doxycycline has no effect in wild-type NIH3T3 cells, transgenic expression of human SMN is able to correct cell proliferation defects in Smn-deficient NIH3T3 cells [11], indicating that the effects are specifically due to SMN depletion. We therefore investigated the effect of *SMN2* gene expression on cell proliferation in NIH3T3-SMN2/Smn_RNAi_ cell lines cultured in the presence or absence of doxycycline for 7 days (Figure 5A). Smn deficiency resulted into a twenty-fold difference in the number of NIH3T3-Smn_RNAi_ cells relative to wild-type NIH3T3 cells, in which doxycycline had no effect on proliferation. Consistent with the expression of low levels of human SMN from the *SMN2* gene, NIH3T3-*SMN2*_low_/SMN_RNAi_ cells exhibited a strong cell proliferation defect following knockdown of endogenous Smn, but this effect was significantly less severe than that in NIH3T3-Smn_RNAi_ cells. In particular, despite very slow cell proliferation, Smn-deficient NIH3T3-SMN2_low_/Smn_RNAi_ cells unlike NIH3T3-Smn_RNAi_ cells did not stop growing even after prolonged culture in the presence of doxycycline. Increased levels of human SMN expression from the *SMN2* gene in NIH3T3-SMN2_high_/Smn_RNAi_ cells resulted in a robust albeit incomplete correction of the cell proliferation phenotype triggered by Smn deficiency, with NIH3T3-SMN2_high_/Smn_RNAi_ cells on average cycling ∼10% more slowly than wild-type NIH3T3 cells in the presence of doxycycline. These results indicated that proliferation of Smn-deficient NIH3T3-SMN2/Smn_RNAi_ cells is proportional to the levels of human SMN expressed from the *SMN2* gene.

Next, we investigated SMN function and its correlation with proliferation in NIH3T3-SMN2/Smn_RNAi_ cell lines. The only molecularly defined activity of SMN is in the assembly of the Sm core on spliceosomal snRNAs [53], [54] and the degree of reduction in snRNP assembly correlates with disease severity in mouse models of SMA [7]. We previously showed that snRNP assembly is severely decreased in Smn-deficient NIH3T3-Smn_RNAi_ cells and restored by expression of human SMN, while doxycycline has no effect [11]. We carried out *in vitro* snRNP assembly experiments using radioactive U1 snRNA and extracts from NIH3T3 cell lines cultured with or without doxycycline followed by immunoprecipitation with anti-SmB antibodies to monitor Sm core formation *in vitro*. These experiments showed that the SMN-dependent snRNP assembly defects are slightly (two-fold) less severe in NIH3T3-SMN2_low_/Smn_RNAi_ cells compared to NIH3T3-Smn_RNAi_ cells that do not contain the *SMN2* gene and significantly rescued in NIH3T3-SMN2_high_/Smn_RNAi_ cells (Figure 5B). Overall, these data demonstrated that SMN expression from the *SMN2* gene correlates well with snRNP assembly activity and cell proliferation in our model system.

### Development of a Cell-based Phenotypic Assay for SMN-dependent Cell Proliferation in NIH3T3 Cells

**Figure 6:**
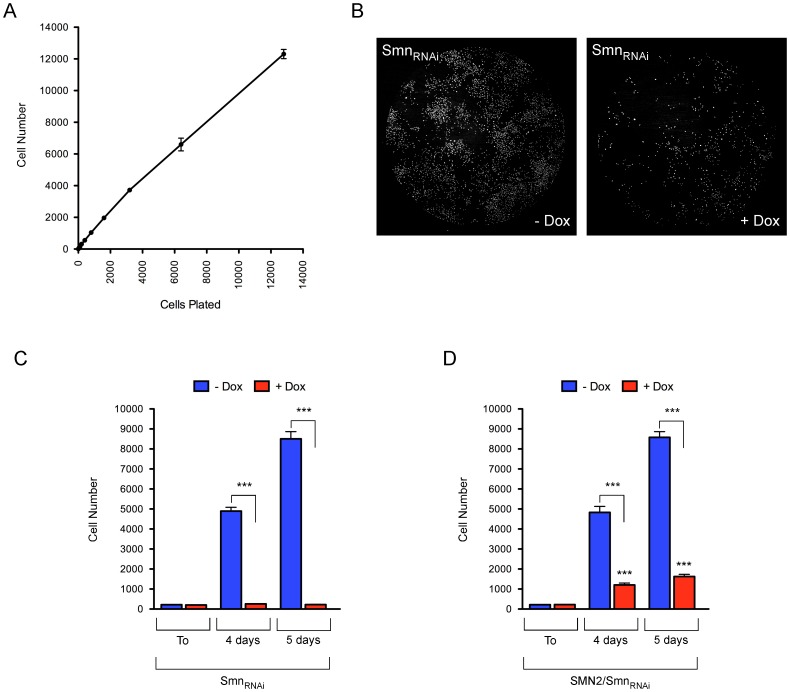
An SMN-dependent cell proliferation assay in 96-well format. (A) Automated determination of NIH3T3 cell number in 96-well format. Two-fold serial dilutions of NIH3T3-SMN2_low_/SMN_RNAi_ cells were plated in eight replicate wells of a 96-well plate. Following fixation and Hoechst staining 4 hours later, cell number was determined by imaging whole wells with an IN Cell Analyzer 2000. (B) Representative IN Cell Analyzer images of NIH3T3-Smn_RNAi_ cells cultured with or without Dox in a 96-well format. (C–D) Analysis of SMN-dependent cell proliferation in NIH3T3-Smn_RNAi_ (C) and NIH3T3-SMN2_low_/Smn_RNAi_ (D) cells using the 96-well format assay. NIH3T3 cells were cultured for 5 days with or without Dox, and then seeded in six replicate wells of a 96-well plate. Dox-treatment was continued throughout the experiment. Cell number was determined at 4 hours (T0), 4 days and 5 days post-plating. Data are represented as mean and SEM (n = 6; *** = p<0.001; two-way ANOVA).

We sought to develop a cell-based assay for high-throughput chemical and genetic screens of modifiers of SMN expression and function that uses reduced proliferation as a robust phenotypic readout of SMN deficiency in NIH3T3 cells. First, we developed an automated, imaging-based approach to determine cell number in 96-well format as a measure of SMN-dependent cell proliferation in NIH3T3 cells. Serial dilutions of NIH3T3-SMN2_low_/Smn_RNAi_ cells were seeded in a 96-well optical plate, followed by fixation and nuclear staining with Hoechst 4 hours later. Direct determination of cell number was then carried out by whole well imaging with an IN Cell Analyzer. These experiments demonstrated a linear relationship between the number of cells plated and the optical readout of Hoechst-stained nuclei (Figure 6A). Thus, in addition to being rapid and cost-effective, this methodology showed remarkable linearity over a wide range of cell number.

Next, we investigated whether SMN-dependent effects on cell proliferation in NIH3T3 cells could be assessed using a 96-well format and the above readout. To do so, NIH3T3-Smn_RNAi_ and NIH3T3-SMN2_low_/Smn_RNAi_ cells were cultured with and without doxycycline for 5 days prior to seeding 200 cells into 6 replicate wells of a 96-well plate, with doxycycline treatment continuing throughout the course of the experiment. Cell number was determined at 4 hours (time zero) as well as 4 and 5 days post-plating using Hoechst staining followed by imaging with the IN Cell Analyzer. Figure 6B shows representative whole well images of normal and SMN-deficient NIH3T3-SMN2_low_/Smn_RNAi_ cells at 5 days post-plating in 96-well plates. These experiments revealed a similar time-dependent increase in the number of untreated NIH3T3-Smn_RNAi_ and NIH3T3-SMN2_low_/Smn_RNAi_ cells with normal levels of SMN and the time zero analysis confirmed the presence of equal numbers of cells at plating (Figure 6C-D). Consistent with the growth arrest phenotype at the time of plating, the number of doxycycline-treated NIH3T3-Smn_RNAi_ cells did not change over time (Figure 6C). In contrast, doxycycline-treated NIH3T3-SMN2_low_/Smn_RNAi_ showed a modest time-dependent increase in cell number (Figure 6D), consistent with both a much slower rate of proliferation relative to untreated cells and the differential severity of the phenotype triggered by Smn deficiency in NIH3T3-SMN2_low_/Smn_RNAi_ compared to NIH3T3-Smn_RNAi_ cells (see also Figure 5). The largest difference in cell number between normal and Smn-deficient cells was observed at 5 days post-plating for both NIH3T3-Smn_RNAi_ (∼40-fold) and NIH3T3-SMN2_low_/Smn_RNAi_ (∼5-fold) cells. Thus, we established a cell-based phenotypic assay in 96-well format that accurately measures SMN-dependent effects on proliferation of NIH3T3 cells.

### Genetic and Pharmacological Modulation of SMN-dependent Cell Proliferation in NIH3T3 Cells

**Figure 7:**
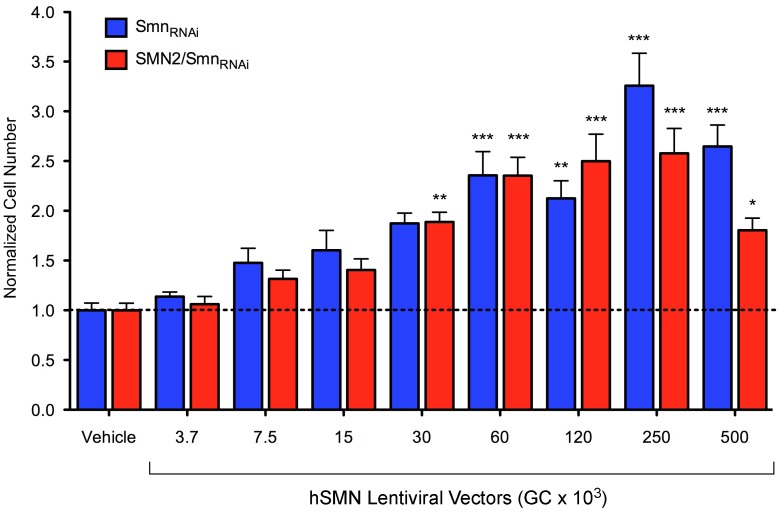
Genetic modulation of SMN-dependent proliferation in NIH3T3 cells. Dose-response analysis of lentiviral-mediated human SMN expression on cell proliferation of SMN-deficient NIH3T3-Smn_RNAi_ and NIH3T3-SMN2_low_/Smn_RNAi_ cells. In these experiments, NIH3T3 cells were cultured for 5 days with Dox prior to seeding in six replicate wells of a 96-well plate in the presence of Dox. Vehicle or increasing amounts of SMN-expressing lentivirus were added 4 hours later. For each group and treatment, cell number was determined at 5 days post-plating and normalized to that of the corresponding vehicle-treated cells. Data are represented as mean and SEM (n = 6; * = p<0.05; ** = p<0.01; *** = p<0.001; one-way ANOVA).

Next we determined whether the cell proliferation defects caused by SMN deficiency in NIH3T3 cells could be modulated by genetic and chemical approaches. First, we investigated the capacity for the system to respond to genetic approaches by assessing the ability of lentiviral-mediated human SMN expression to correct the cell proliferation phenotype in Smn-deficient NIH3T3 cells using our 96-well format assay. Both NIH3T3-Smn_RNAi_ and NIH3T3-SMN2_low_/Smn_RNAi_ cells in which Smn deficiency was induced by preincubation with doxycycline for 5 days were plated in 6 replicate wells of 96-well plates in the presence of doxycycline as well as increasing amounts of lentivirus expressing human SMN driven by the CMV promoter. The number of vehicle- and lentivirus-treated NIH3T3 cells was determined 5 days later using Hoechst nuclear staining and IN Cell Analyzer imaging. These experiments revealed that human SMN expression promoted cell proliferation of Smn-deficient NIH3T3 cells in a dose-dependent manner with a maximum increase in cell number of 3.5-fold for NIH3T3-Smn_RNAi_ cells and nearly 3-fold for NIH3T3-SMN2_low_/Smn_RNAi_ cells compared to their corresponding vehicle-treated controls (Figure 7). With the amount of lentivirus that most effectively promotes cell proliferation, the level of human SMN expressed is equivalent to endogenous mouse Smn in wild-type NIH3T3 cells (data not shown). Furthermore, lentiviral-mediated expression of GFP under the same conditions had no effect on the proliferation of Smn-deficient NIH3T3 cells (data not shown), confirming the specificity for the effects of SMN restoration.

**Figure 8:**
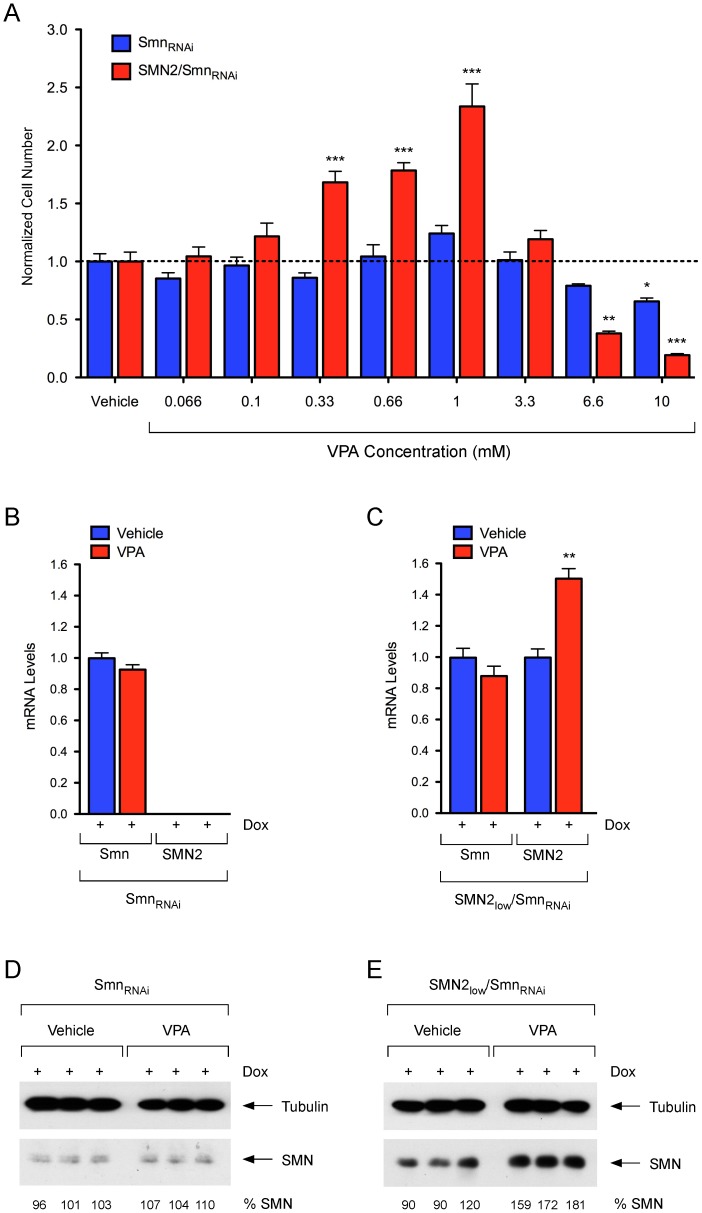
Pharmacological modulation of SMN-dependent proliferation in NIH3T3 cells. (A) Dose-response analysis of VPA treatment on cell proliferation of SMN-deficient NIH3T3-Smn_RNAi_ and NIH3T3-SMN2_low_/Smn_RNAi_ cells. In these experiments, NIH3T3 cells were cultured for 5 days with Dox prior to seeding in six replicate wells of a 96-well plate in the presence of Dox. Vehicle or increasing amounts of VPA were added 4 hours later. For each group and treatment, cell number was determined at 5 days post-plating and normalized to that of the corresponding vehicle-treated cells. Data are represented as mean and SEM (n = 6; * = p<0.05; ** = p<0.01; *** = p<0.001; one-way ANOVA). (B–C) RT-qPCR analysis of mouse Smn and human SMN2 mRNA levels in NIH3T3-Smn_RNAi_ (B) and NIH3T3-SMN2_low_/Smn_RNAi_ (C) cells cultured with Dox for 3 days and then in the presence of either water (Vehicle) or 1 mM VPA for additional 4 days. Data from triplicate RT-qPCR experiments normalized to Gapdh mRNA are represented as mean and SEM (** = p<0.01; t-test). (D–E) Western blot analysis of SMN protein levels in NIH3T3-Smn_RNAi_ (D) and NIH3T3-SMN2_low_/Smn_RNAi_ (E) cells cultured with Dox for 3 days and then in the presence of water (Vehicle) or 1 mM VPA for additional 4 days. Blots were probed with an antibody that recognizes both mouse and human SMN proteins. SMN levels in VPA-treated relative to vehicle-treated cells are shown at the bottom.

We also tested whether SMN-dependent cell proliferation could be modulated by treatment with small chemical compounds. We used the histone deacetylase inhibitor valproic acid (VPA), which was previously demonstrated to increase SMN levels in human SMA fibroblasts [55], [56], leading to improved motor function and survival in a mouse model of SMA [57]. We performed dose-response analysis of VPA effects on cell proliferation of doxycycline-treated NIH3T3-SMN2_low_/Smn_RNAi_ and NIH3T3-Smn_RNAi_ cells using the same conditions employed in lentiviral transduction experiments. We found that VPA treatment resulted in dose-dependent stimulation of cell proliferation in Smn-deficient NIH3T3-SMN2_low_/Smn_RNAi_ cells compared to vehicle-treated controls (Figure 8A). There was a maximum 2.5-fold increase in cell number at 1 mM, while higher drug concentrations appeared toxic. Importantly, VPA treatment had no effect on the proliferation of Smn-deficient NIH3T3-Smn_RNAi_ cells lacking the *SMN2* gene (Figure 8A). The specificity of the effects for NIH3T3-SMN2_low_/Smn_RNAi_ cell lines containing the *SMN2* gene was consistent with the predicted mechanism of action for VPA in increasing *SMN2* gene expression. To determine whether this was indeed the case, we compared SMN mRNA and protein levels in Smn-deficient NIH3T3-SMN2_low_/Smn_RNAi_ and NIH3T3-Smn_RNAi_ cells treated with 1 mM VPA relative to vehicle-treated controls. RT-qPCR experiments showed that VPA treatment did not change the low level of Smn mRNA in either NIH3T3-SMN2_low_/Smn_RNAi_ or NIH3T3-Smn_RNAi_ cells (Figure 8B–C), indicating that it did not interfere with the doxycycline-dependent RNAi knockdown of endogenous mouse Smn. In contrast, VPA treatment resulted in a ∼1.5-fold increase in total SMN mRNA produced from the *SMN2* gene in NIH3T3-SMN2_low_/Smn_RNAi_ cells compared to vehicle-treated cells (Figure 8C). A similar increase was also found for full-length SMN mRNA (data not shown). In agreement with the observed changes in mRNA levels, Western blot analysis showed that the low Smn protein levels were unaffected by VPA in Smn-deficient NIH3T3-Smn_RNAi_ cells (Figure 8D), while VPA treatment increased SMN protein levels (1.7-fold, p<0.01) in NIH3T3-SMN2_low_/Smn_RNAi_ cells compared to vehicle-treated cells (Figure 8E). These results were consistent with the beneficial effects of VPA on proliferation of NIH3T3-SMN2_low_/Smn_RNAi_ cells being the consequence of SMN upregulation. The observation that moderate SMN induction with VPA elicits an effect on cell proliferation of NIH3T3-SMN2_low_/Smn_RNAi_ cells similar to that of complete, lentiviral-mediated SMN restoration can be explained by differences in the timing of SMN expression. Characteristic of the lentiviral system, there is a lag of approximately one day between viral transduction and the onset of transgenic SMN expression, which delays the biological effect of SMN restoration. In contrast, small molecules such as VPA can readily engage their cellular targets and act more quickly. It is therefore possible, if not likely that some chemical compounds could have a more robust effect than we observe with the SMN lentivirus in our cell proliferation assay.

Collectively, these experiments provided proof of concept that cell proliferation defects induced by Smn deficiency could be improved by genetic or pharmacological upregulation of SMN in NIH3T3-SMN2_low_/Smn_RNAi_ cells and thus used as a phenotypic readout in future screens for modifiers of *SMN2* gene expression or function.

## Discussion

SMA is a devastating neurological disease for which no effective treatment is currently available. Progress in the discovery of new SMA therapeutic approaches has been hampered, at least in part, by the relatively limited knowledge of targets and the lack of assays in high-throughput format that provide a functional readout of SMN activity. To facilitate the identification of novel targets and therapeutics that act not only on SMN expression but also on SMN function and downstream events induced by SMN deficiency, we developed a cell-based system that uses cell proliferation defects triggered by SMN deficiency in cultured mammalian cells as phenotypic readout of the functional levels of SMN produced from the *SMN2* gene. Following miniaturization of this cell-based assay for use in high-throughput format, we provide proof of principle for the application of this novel platform for unbiased, genetic and chemical screens that aim to identify and characterize modifiers of SMN expression and function that act through any one of multiple, possible mechanisms of action. Thus, our work establishes a powerful new discovery tool for the study of SMN biology and possibly the development of novel approaches for SMA therapy.

SMN is an essential gene that is required for cell growth and viability from yeast to mammals [11], [58]–[62]. The model system we developed is based on a mouse NIH3T3 fibroblast cell line (NIH3T3-Smn_RNAi_) in which regulated knockdown of endogenous mouse Smn triggers a severe cell proliferation defect [11], providing a direct phenotypic readout of SMN function. The rationale of the work was to adapt this system for purposes of therapeutic and biological discovery in SMA research by introducing the entire human *SMN2* gene into NIH3T3-Smn_RNAi_ cells, thereby making cell proliferation dependent on the levels of functional SMN produced from the *SMN2* gene. We successfully isolated NIH3T3 cell lines with either low or high *SMN2* copy numbers and demonstrated that cell proliferation is proportional to the levels of human SMN expression following RNAi depletion of endogenous Smn. Consistent with the phenotypic effects of varying *SMN2* copy numbers in mice lacking the Smn alleles [51], [63], we found that low *SMN2* copy number modestly improves the proliferation phenotype of Smn-deficient NIH3T3 cells, while high *SMN2* copy number is remarkably protective. In animal models of SMA, snRNP assembly defects correlate with disease severity [7] and restoration of snRNP levels coincides with phenotypic correction [8], [64]. Similarly, we show a direct correlation between the degree of SMN-dependent snRNP assembly impairment and the severity of the cell proliferation phenotype in our model system. Moreover, functional analysis of several SMN point mutations associated with SMA revealed that their ability to support snRNP assembly and correct the proliferation defects in Smn-deficient NIH3T3 fibroblasts is proportional to their potency in rescuing motor neuron phenotypes in animal models and inversely correlated with clinical severity in patients (L. Pellizzoni, C. Beattie and A. Burghes, unpublished results). Lastly, reduced cell proliferation induced by SMN deficiency in select brain regions has been associated with impaired perinatal brain development in severe SMA mice [65]. Altogether, this evidence supports the conclusion that the effect of SMN deficiency on cell proliferation in this system mirrors at least some of the mechanisms at play in the disease.

As the copy number of the nearly identical *SMN2* gene correlates with disease severity [21]–[23], SMA therapeutic development has primarily focused on enhancing the expression of full-length SMN from the *SMN2* gene or through gene therapy [24]–[27]. To date, cell-based screens for SMA therapeutic discovery have employed reporter assays that monitor increased *SMN2* transcription and exon 7 splicing [36]–[39]. Immunodetection and imaging methodologies were also used to screen for small molecules that upregulate SMN protein levels in human SMA fibroblasts [40]. However, cell-based phenotypic assays that measure increased SMN expression and function from the *SMN2* gene and are amenable to high-throughput screening have not been developed. Thus, our cell system has a number of distinct benefits, which complement and significantly advance currently available tools for SMA translational research, affording the opportunity to identify cellular factors and genetic networks linked to SMN biology in a comprehensive and mechanistically unbiased manner. First, the presence of the entire *SMN2* gene allows for the identification of potential modifiers acting at any level of SMN gene expression from transcription to protein turnover. Second, our platform is able to capture the possible beneficial effects of modifiers that act not only on SMN expression but also on its activity as well as downstream SMN-dependent pathways. Lastly, comparison of the effects of candidate chemical and genetic modifiers on the proliferation of SMN-deficient NIH3T3-Smn_RNAi_ and NIH3T3-SMN2/Smn_RNAi_ cell lines will provide the opportunity for effective hit deconvolution and rapid identification of modifiers acting through SMN2-dependent and -independent mechanisms for priority ranking and early mechanistic insights.

The robust cell proliferation phenotype of NIH3T3-SMN2/Smn_RNAi_ cells combined with the development of an assay in 96-well format provides the foundation for the applicability of our platform to high-throughput screening. Additionally, direct measure of NIH3T3 cell number by a single-step method comprising nuclear Hoescht staining followed by automated imaging is a rapid, cost-effective and accurate method for assessing SMN-dependent cell proliferation in the 96-well format. In support of the conclusion that our phenotypic platform is suitable for screening modifiers of SMN biology, we provide proof-of-concept that it can respond to either genetic or pharmacological intervention. We show that lentiviral-mediated SMN transduction promotes proliferation of both NIH3T3-SMN2/Smn_RNAi_ and NIH3T3-Smn_RNAi_ cells in a dose-dependent and SMN2-independent manner, highlighting the specificity and reversibility of the phenotype induced by Smn deficiency. Conversely, consistent with *SMN2* gene upregulation and the results of previous studies [55], [56], we show that the beneficial effects of treatment with the HDAC inhibitor VPA on cell proliferation are restricted to Smn-deficient NIH3T3-cells containing *SMN2*. Together, these features support the viability of our system for use in high-throughput chemical and genetic screening projects for SMA discovery.

In conclusion, the cell system for phenotypic screening we developed provides a powerful new tool for identifying and validating modifiers of SMN expression and function, which promise to yield not only critical insights into our understanding of the basic biology of SMN but also new therapeutic avenues for the treatment of SMA.

## Materials and Methods

### NIH3T3 Cell Lines and Tissue Culture

The NIH3T3 cell line with inducible knockdown of endogenous mouse Smn (NIH3T3-Smn_RNAi_) used in this study has been described previously [11], [12]. To generate human *SMN2*-containing NIH3T3 cell lines with regulated Smn knockdown, the 35.5 kb BamHI fragment corresponding to the human genomic region encompassing the *SMN2* gene, previously used to generate SMA transgenic mice [51], was excised from a BAC construct (a gift from Dr. Arthur Burghes) and cloned into the SuperCos1 cosmid vector (Stratagene). The resulting vector was transfected into NIH3T3-Smn_RNAi_ cells and stable NIH3T3-SMN2/Smn_RNAi_ cells were isolated through antibiotic selection with G418 (0.5 mg/ml). Clonal cell lines were obtained by limiting dilution in 96-well plates.

NIH3T3 cells were cultured in Dulbecco’s Modified Eagle Medium (DMEM) with high glucose (Gibco) containing 10% fetal bovine serum (HyClone), 2 mM glutamine (Gibco), and 0.1 mg/ml gentamicin (Gibco). Smn RNAi was induced by addition of doxycycline (Fisher) to the growth medium at a final concentration of 100 ng/ml as previously described [11], [12].

Lentiviral constructs expressing GFP and SMN driven by the CMV promoter were generated by standard cloning techniques using the pRRLSIN.cPPT.PGK-GFP.WPRE vector (Addgene plasmid 12252) as a backbone [66], [67]. For lentivirus production, viral stocks pseudotyped with the vesicular stomatitis G protein (VSV-G) were prepared by transient co-transfection of 293 T cells as previously described [11]. Lentiviral particles in supernatants were harvested, concentrated by ultracentrifugation for 2 hours and 30 minutes at 19,500 rpm in a SW28 rotor (Beckman), and titered using the Lenti-X™ qRT-PCR Titration Kit (Clontech).

### DNA and RNA Analysis

For genomic DNA analysis, cells were washed twice in ice cold PBS and harvested into 500 µl of PK buffer (10 mM EDTA, 100 mM Tris-HCl, pH 7.5, 300 mM NaCl, 2% SDS) containing 1 mg/ml proteinase K followed by overnight incubation at 50°C with shaking at 800 rpm. After addition of 500 µl of isopropanol and further incubation for 2 hours at room temperature, genomic DNA was pulled out of solution and resuspended in TE buffer (10 mM Tris-HCl, 1 mM EDTA pH 7.5).

Total RNA from NIH3T3 cells was isolated using Trizol reagent (Invitrogen) followed by digestion with DNase I (Ambion). RNA (1 µg) was reverse transcribed using RevertAid First Strand cDNA Kit (Fermentas). For analysis of SMN2 exon 7 splicing, semi-quantitative RT-PCR reactions were carried out in the presence of a forward primer 5′ end-labeled using T4 polynucleotide kinase (New England BioLabs) and [^32^P]-ATP (Perkin Elmer) as previously described [12]. PCR products were amplified using AmpliTaq Gold DNA polymerase (Roche), resolved on an 8% polyacrylamide-8 M urea gel, and subjected to autoradiography. The linear range of PCR amplication was determined independently for each experiment. Quantification of radiolabeled PCR products was carried out using a Typhoon PhosphorImager (Molecular Dynamics). For quantitative RT-qPCR experiments, triplicate reactions were carried out using Power SYBR Green PCR master mix (Applied Biosystem) in a Realplex^4^ Mastercycler (Eppendorf). RT-qPCR data were normalized to Gapdh mRNA. The primers utilized in this study were previously reported [12].

### Antibodies

The following antibodies were used in this study: anti-SMN clone 8 (BD Transduction Laboratories), anti-SMN 7F3 [68], anti-SmB 18F6 [69], anti-human SMN (a gift from Dr. Adrian Krainer), and anti-Tubulin DM 1A (Sigma).

### Western Blot Analysis

Analysis was performed as described previously [12]. In short, proteins were fractionated by SDS/PAGE on 12% polyacrylamide gels and transferred onto a Trans-Blot transfer medium nitrocellulose membrane (Bio-Rad) using 1X Tris-glycine buffer (Bio-Rad) containing 20% methanol. The blots were blocked in 5% nonfat dry milk in PBS containing 0.1% Tween 20 for 1 h at room temperature. Membranes were washed 3 times for 10 minutes with PBS containing 0.1% Tween 20 at room temperature. Incubation with secondary antibodies conjugated to horseradish peroxidase was performed in PBS containing 0.1% Tween 20 for 1 h at room temperature. Chemiluminescence was carried out using a Supersignal West Pico Chemilumiescent substrate according to the supplier’s recommendations (Thermo Scientific). SMN protein levels were quantified after normalization to Tubulin using ImageJ.

### Immunofluorescence Analysis

NIH3T3 cells were grown on glass coverslips in 24-well plates. Cells were washed once with PBS and then fixed in 4% paraformaldehyde-PBS for 15 minutes at room temperature. Following fixation, cells were permeabilized in 0.5% Triton-X in PBS for 5 minutes at room temperature. Blocking and both primary and secondary antibody incubations were performed with 3% BSA in PBS. Images were collected with a SP5 confocal microscope (Leica).

### In vitro snRNP Assembly

*In vitro* snRNP assembly experiments were performed using radioactive U1 snRNA and NIH3T3 cell extracts (25 µg) followed by immunoprecipitation with anti-SmB antibodies, electrophoresis on denaturing polyacrylamide gels and autoradiography as previously described [7], [11]. Quantification of immunoprecipitated U1 snRNA was carried out using a Typhoon PhosphorImager (Molecular Dynamics).

### Cell Proliferation Assay in 96-well Format

NIH3T3 cells were cultured with or without doxycycline for 5 days prior to seeding in 96-well format. NIH3T3 cells were counted with an automatic digital cell counter (ADAM, Digital Bio) and dispensed in 96-well optical plates (Grainer) at 200 cells per well in 200 µl of media continuing the prior treatment with or without doxycycline. Vehicle and serial dilutions of valproic acid or lentiviral particles were added 4 hours post-plating (six replicate wells for each concentration) and NIH3T3 cells further incubated for different times in culture. For determination of cell number, a 4X solution containing 16% paraformaldehyde and 8µg/ml of Hoechst in PBS was added to each well of the 96-well plate and incubated for 15 minutes at room temperature. After washing with PBS, whole-well imaging acquisition was performed with the IN Cell Analyzer 2000 (GE Healthcare) followed by nuclear counting with the IN Cell Investigator (GE Healthcare) software.

### Statistical Analysis

Statistical analysis was carried out with the Prism 5 (GraphPad) software using two-tailed unpaired Student’s t-test and one-way or two-way ANOVA followed by the Bonferroni post-hoc test as indicated. Data are represented as mean plus SEM and P values are indicated as follows: * = p<0.05; ** = p<0.01; *** = p<0.001.
